# Proceedings of the Medical Association of the State of Alabama

**Published:** 1853-05

**Authors:** 


*Proceedings of the Medical Association of the State of Alabama, at its Sixth Annual Meeting, held in the city of Selma.* 1852.

We presume that our readers are not indifferent to the efforts made in different parts of our country to advance medical knowledge, and improve the character of the profession. They are doubtless glad to be informed of what others are doing for these ends, as a mere matter of medical intelligence. But the advantage, it may be hoped, will extend farther than this. Such information is well calculated to afford encouragement, and develop a spirit of emulation in undertaking, or carrying out more zealously enterprises of a similar character. In proportion as these effects follow, good will be accomplished.

These reflections have arisen on taking up for notice the work with the above title. It is a volume of one hundred and sixty-eight pages. We have before had occasion to notice former volumes of Transactions emanating from this society. The present publication shows that the society continues to flourish. As a whole the papers contained in the present volume are highly creditable. We have only space to give the table of contents:

"Report of the Diseases of Mobile, by W. H. Anderson, M. D., and G. A. Ketchum, M. D.; Report on Indigenous Botany of Clarke, by A. Denny; Report on the Diseases of Centreville and vicinity, by J. W. Crawford, M.D.; Report on Surgery, by L. H. Anderson, M. D.; Cases reported by J. T. Gee, M. D., Burnsville; Report on the Article Gelseminum Sempervirens, by J. C. Batchelor, M. D., of Dallas county; Report on Diseases of Cahaba, by J. A. English, M. D.; Report on Indigenous Botany of Perry, by F. A. Bates, M. D.; Changeability of Disease, by W. Taylor, M. D., of Talladega; Case of Dislocation of Spleen, by J. C. Batchelor, M. D.; Paper on the Unity of Disease, by H. Backus, M. D., Selma; Prize Essay on the Summer and Autumnal Fevers of South Alabama, to which is appended, some remarks on the Diagnosis and Treatment of Typhoid Fever, by L. H. Anderson, M. D., Sumter county; Valedictory Address, by F. A. Bates, M. D., Marion; Reports on the Number and Character, &c., of Practitioners of Medicine, by J. P. Barnes, M. D., Mobile, A. Denny, M. D., Clarke, F. E. Gordon, M. D., Marion, and R. H. Erwin, M. D., Camden; Names of Members of the Association."

---

*What is Man?*—We find in one of our exchanges the following answer to this question: "Chemically speaking, a man is forty-five pounds of carbon and nitrogen, diffused through five and a half pails full of water!" This is more in the spirit of modern science than the ancient definition of Plato, or the modern description by Carlyle.

